# Bullet screen in pre-clinical undergraduate pharmacology education: a survey study

**DOI:** 10.1186/s12909-022-03906-6

**Published:** 2022-11-28

**Authors:** Yaoxing Chen, Hong Qi, Yu Qiu, Juan Li, Liang Zhu, Hao Wang, Xiaoling Gao, Gan Jiang

**Affiliations:** Department of Pharmacology and Chemical Biology, Shanghai Universities Collaborative Innovation Center for Translational Medicine, Shanghai Jiao Tong University School of Medicine, 280 South Chongqing Road, Shanghai, 200025 China

**Keywords:** Bullet screen, Pharmacology education, Pre‑clinical undergraduate, Survey study

## Abstract

**Background:**

The lack of interaction and communication in pharmacology courses, especially since the onset of the coronavirus disease 2019 (COVID-19) pandemic, which required a fast shift to remote learning at medical schools, leads to an unsatisfactory learning outcome. New interactive teaching approaches are required to improve pharmacology learning attention and interaction in remote education and traditional classrooms.

**Methods:**

We introduced bullet screens to pharmacology teaching. Then, a survey was distributed to first-, second- and third-year pre-clinical undergraduate medical and nursing students at the Shanghai Jiao Tong University School of Medicine from November 2020 to March 2022. We evaluated the essential features, instructional effectiveness, and entertainment value of bullet screens. Responses to structured and open-ended questions about the strengths and weaknesses of the bullet screen and overall thoughts were coded and compared between medical and nursing students.

**Results:**

In terms of essential features, bullet screens have a high degree of acceptability among students, and this novel instructional style conveniently increased classroom interaction. Considering instructional effectiveness, bullet screen may stimulate students’ in-depth thinking. Meanwhile, students tended to use bullet-screen comments as a way to express their support rather than to make additional comments or to express their different viewpoints. The entertainment value of bullet screen was noteworthy. The lack of ideas might lead to relative differences between medical and nursing students, indicating that guiding the appropriate use of bullet screen is necessary.

**Conclusions:**

The bullet screen may be popularized as an auxiliary teaching approach to promote interaction between teachers and students in the classroom as well as during remote education. It is an interesting and beneficial tool in pharmacology courses, yet there are several aspects of this device that should be improved for popularization.

**Supplementary Information:**

The online version contains supplementary material available at 10.1186/s12909-022-03906-6.

## Background

Pharmacology teaching contributes to various majors, including clinical medicine, nursing, dentistry, pharmacy, etc. In medical education, a thorough understanding of pharmacology is needed for effective pharmacotherapy and the prescribing of medication [1]. However, pharmacology is complex and contains considerable theoretical knowledge, including the pharmacological mechanism and action, clinical application and adverse reactions. Course content is massive and abstract. Medical students identified that they needed more pharmacology teaching in their curriculum [2, 3]. Additionally, in nursing, pharmacology training has been claimed to be insufficient [4, 5]. Therefore, current pharmacology delivery requires further optimization to achieve better learning outcomes. The involvement of active teaching styles with collaborative learning can lead to excitement of the exchange of ideas and thus achieve a better learning outcome compared to the traditional format with passive lectures [6]. In our study, we attempted to introduce bullet screen into the classroom to improve classroom interaction and thus improve students’ learning outcomes.

Bullet screen is a technology that allows real-time comments from viewers to fly across the screen like bullets. The great majority of Chinese college students prefer watching films with bullet-screen remarks because of their subculture context, ease of use, high immediacy, and, most importantly, “social viewing” characteristics [7]. Young people feel that the bullet screen will encourage their self-expression and self-identification. These characteristics of bullet screen correspond well with the needs of educational reform [8, 9]. However, bullet screen technology has seldom been applied in medical education. When posting bullet screen comments, students have the illusion of commenting with other audience members at the same time. With the help of the WeChat application, the bullet screen can be utilized by scanning the Quick-Response code and sending a text. Then the real-time bullet screen comments will appear on top of the slides. The privacy of students is also protected, thus encouraging them to contribute. Therefore, as an auxiliary teaching tool, bullet screen is simple and convenient, and can improve students’ engagement. We supposed that using bullet screen would improve student interaction. This may have a positive effect on student learning as active learning strategies which have been shown to enhance student learning experiences [10–12].

We used bullet screens as an interactive platform to ask medical students entertaining and thought-provoking questions within a pharmacology course. We introduced clinical cases into pharmacology education and encouraged students to think about the patient’s condition and rational drug use. We also introduced questions on the keynotes and error-prone points. In addition, we guided students to explore the advanced knowledge of pharmacology. We also used multiple-choice questions as consolidation exercises. To estimate the acceptance among students, their opinions, and the effect of bullet screen on teaching and classroom interaction, we designed this survey study. We also compared the difference in its application among students majoring in nursing and clinical medicine. From the results of this investigation, we also proposed recommendations for the improvement of bullet screen in pharmacology education.

## Methods

### Study population

This study was conducted at the Shanghai Jiao Tong University School of Medicine. Eligible participants included medical and nursing students enrolled in their first, second or third year as from November 2020 to March 2022. Our study took place both in online learning and in a face-to-face classroom. The Ethics Committee of Shanghai Jiao Tong university School of Medicine approved this protocol. We designed a web-based questionnaire with www.wjx.cn (questionnaire created on November 10th 2020). The questionnaire link was shared in the PowerPoint during class. All the students were invited to complete the questionnaire once after participating in the bullet screen classroom for the first time. The survey was voluntary. In the classroom with bullet screen, 310 medical students and 70 nursing students participated. The questionnaire was completed by 127 students, with the data of 16 students being excluded since no bullet screen was used by these students. A 33.4% response rate was achieved.

### Survey design and implementation

We designed a total of 17 questions, and only one correct answer was acceptable. Five points were assigned for “strongly agree”, 4 points were assigned for “agree”, 3 points were assigned for “neutral”, 2 points were assigned for “disagree” and 1 point was assigned for “strongly disagree”. To compute the predicted score for each question, we used the average value. Bullet screens were examined based on their fundamental characteristics, learning outcomes, and entertainment appeal. The Supplementary Appendix File contains the questionnaire.

### Statistical analyses

We considered the average value to calculate the expected score for each question. Descriptive statistics were generated using the mean and standard deviations or counts/frequencies where appropriate, using IBM SPSS Statistics software (version 22.0; IBM Corp., Armonk, NY, USA). To assess internal consistency reliability for structured/closed-ended survey items graded on the same Likert scale, Cronbach’s alpha calculations were also performed using IBM SPSS Statistics software (version 22.0; IBM Corp., Armonk, NY, USA). The statistical tests and percent stacked bar chart were performed with R 4.1.3 (http://www.r-project.org/).

### Ethical considerations

We have taken two primary courses of action to ensure that students felt free to share feedback honestly without fear of repercussions. This survey was completely anonymous, with no student names or identifying information submitted. Also, the survey was voluntary. Data collectors had no way to know who had completed it or who had not. Students were informed at the time of distribution that the survey was both anonymous and optional. We also informed students that their responses would not be linked to their names or course evaluations.

## Results

Between November 2020 and March 2022, 111 students participated in this bullet screen classroom. The Cronbach’s alpha for the survey was 0.85 (95% confidence interval 0.8 to 0.9), and the Kaiser-Meyer-Olkin (KMO) value was 0.81 (*P* < 0.001), indicating that this survey had high validity and reliability. There were 25 male participants (22.5%) and 86 female participants (77.5%). Table 1 illustrates the general demographic features. Fifty-five (49.6%) of the 111 students majored in clinical medicine, while 56 (50.5%) majored in nursing. There were 4 (3.6%) first-year students, 81 (73.0%) second-year students, and 26 (23.4%) third-year students among those who responded. Eighty-eight students (79.3%) were between the ages of 18 and 20, with the remainder between the ages of 21 and 23.

**Table 1:** Demographic characteristics of participants

	No. of Study Participants	Major in Clinical Medicine	Major in Nursing
Overall	111	55 (49.6%)	56 (50.5%)
Sex			
Men	25 (22.5%)	22 (19.8%)	3 (2.7%)
Women	86 (77.5%)	33 (29.7%)	53 (47.8%)
Age groups, y			
18–20	88 (79.3%)	32 (28.8%)	56 (50.5%)
21–23	23 (20.7%)	23 (20.7%)	0 (0.0%)
Grade			
First-year	4 (3.6%)	4 (3.6%)	0 (0.0%)
Second-year	81 (73.0%)	25 (22.5%)	56 (50.5%)
Third-year	26 (23.4%)	26 (23.4%)	0 (0.0%)

### The general consensus benefits of the bullet screen classroom

**Fig. 1:**
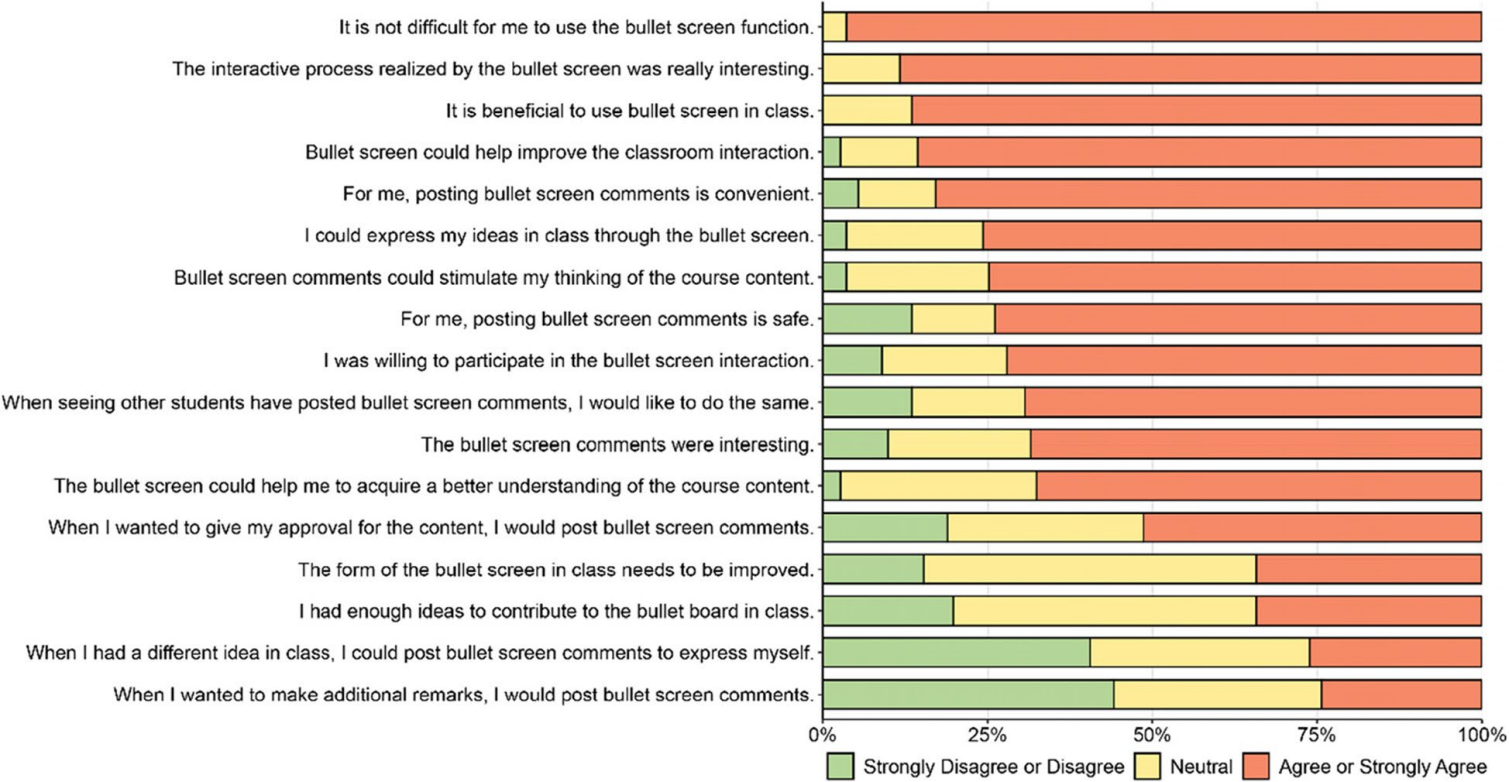
Perceptions regarding the value of bullet screen among pre-clinical students at the University of Shanghai Jiao Tong University, School of Medicine. Students were asked to rate their level of agreement with a total of 17 questions, and one answer was acceptable

According to the findings of the study, the bullet screen classroom had a high degree of acceptability among 111 pre-clinical students, and this novel instructional style may assist in increasing classroom interaction (Fig. 1). Because of the popularity of bullet screen interaction among Chinese college students, almost all of them (107/111, 96.4%) agreed or strongly agreed that it was not difficult for them to utilize the bullet screen function in class, and this format was convenient for 92 (82.9%) of them. This approach was useful for the majority of students (96/111, 86.5%). The interactive procedure enabled by the bullet screen was considered highly interesting by 98 (88.3%) students. A total of 95 students (86.5%) agreed or strongly agreed that that using a bullet screen may increase classroom engagement. A total of 84 students (75.7%) agreed or strongly agreed that bullet-screen remarks are a useful approach to expressing themselves in class. Furthermore, 83 (74.8%) of students agreed or strongly agreed that engagement via the bullet screen inspired their in-depth thinking in class. All of the above seven traits were averaged out to illustrate the positive aspects, which all scored more than 4 (Table 2). These results showed that bullet screen has a very wide popularization and application prospects.

**Table 2:** The overall scores for each question, ranked by scores

Questionnaire	Score (Mean ± s.d.)^*^
It is not difficult for me to use the bullet screen function.	4.6 ± 0.6
The interactive process realized by the bullet screen was really interesting.	4.3 ± 0.7
Bullet screen could help improve the classroom interaction.	4.2 ± 0.7
It is beneficial to use bullet screen in class.	4.1 ± 0.6
For me, posting bullet screen comments is convenient.	4.1 ± 0.8
I could express my ideas in class through the bullet screen.	4.1 ± 0.8
Bullet screen comments could stimulate my thinking of the course content.	4.0 ± 0.8
For me, posting bullet screen comments is privacy-preserving.	3.8 ± 1.0
I was willing to participate in the bullet screen interaction.	3.8 ± 0.9
When seeing other students have posted bullet screen comments, I would like to do the same.	3.8 ± 1.1
The bullet screen comments were interesting.	3.9 ± 0.9
The bullet screen could help me to acquire a better understanding of the course content.	3.9 ± 0.9
When I wanted to give my approval for the content, I would post bullet screen comments.	3.5 ± 1.0
The form of the bullet screen in class needs to be improved.	3.3 ± 0.8
I had enough ideas to contribute to the bullet board in class.	3.3 ± 1.0
When I had a different idea in class, I could post bullet screen comments to express myself.	2.9 ± 1.0
When I wanted to make additional remarks, I would post bullet screen comments.	2.9 ± 1.0

*Items scoring ≥4 were considered as positive aspects, while the remaining survey items were considered as neutral or negative aspects

The other ten questions among the 111 students revealed a neutral or negative attitude. A total of 15 students (13.5%) were concerned about the bullet screen’s privacy. Even if other students used the bullet screen in the classroom, thirty-one students (31.5%) expressed neutral or negative willingness to use it. For approximately 32% (35/111, 31.5%) of students, the contents of the bullet screen were not entertaining or useful for learning. Only 38 students (34.23%) agreed that they had enough ideas to contribute to the bullet board in class. When having a different idea in class, twenty-nine students (26.13%) would post bullet screen comments to express themselves. When they wanted to make additional remarks, only 27 students (24.32%) would post bullet screen comments.

### Perspectives on the basic properties of bullet-screen interaction in medical and nursing students

Pharmacology is also important for nursing students, but method of instruction is often ignored [13, 14]. This inadequate teaching and a subsequent poor understanding of scientific disciplines cause many nurses to experience difficulties managing patients’ medication or communicating knowledge of medication to patients [14]. Therefore, we tried to apply this bullet screen tool to students majoring in nursing to evaluate its effect. In addition, previous studies found that nursing students had lower mean scores in several learning strategies, such as peer learning, critical thinking, and time and study environment management, compared with medical students [15]. Therefore, we also hope to evaluate the learning outcomes with bullet screen among nursing students and compared the results with medical students, which could guide us in finding aspects that require improvement.

Our study found that the students majoring in nursing were more reluctant to write bullet-screen comments than those majoring in clinical medicine (3.7 ± 0.9 Vs. 4.1 ± 0.9, *P* = 0.02) (Table 3). In addition, the classroom environment is less active in the nursing specialty. When they noticed that other students had written bullet screen comments, fewer nursing students wanted to do the same (3.6 ± 1.2 Vs. 4.1 ± 0.9, *P* = 0.01) (Table 3). Although students appreciated using the message board to express themselves, we discovered that students majoring in both nursing and clinical medicine did not have enough ideas to contribute to the bullet screen in class, and students majoring in nursing were even less forthcoming (Nursing 3.0 ± 0.9 Vs. Clinical medicine 3.5 ± 0.9, *P* = 0.003) (Table 3). For example, teachers asked some open-ended questions, such as, how to improve the application of insulin, whether the application of angiotensin-converting enzyme inhibitors would influence the prognosis of COVID-19 infection, whether heart transplantation from pig could solve the problem of end-stage heart failure. Some students could not think independently and give their own opinions. Students majoring in clinical medicine could put forward more opinions than students majoring in nursing. According to the results, many students had concerns about the privacy of the bullet screen (Table 3). This finding may be attributed to the limited condition of the current bullet screen software, which did not conceal profile pictures. Many students found that the exposure of their personal profile photos made them feel unmasked, because other students could predict the commenter by viewing their profile photo.

**Table 3:** Perspectives on the basic properties of bullet-screen interaction in medical and nursing students

	Major	Number	Mean ± s.d.	*P* value
It is not difficult for me to use the bullet screen function.	Clinical medicine	55	4.8 ± 0.5	0.13
	Nursing	56	4.6 ± 0.6	
For me, posting bullet screen comments is convenient.	Clinical medicine	55	4.2 ± 0.7	0.12
	Nursing	56	4.0 ± 0.9	
It is beneficial to use bullet screen in class.	Clinical medicine	55	4.2 ± 0.6	0.20
	Nursing	56	4.0 ± 0.6	
I could express my ideas in class through the bullet screen.	Clinical medicine	55	4.1 ± 0.9	0.82
	Nursing	56	4.1 ± 0.8	
I was willing to participate in the bullet screen interaction.	Clinical medicine	55	4.1 ± 0.9	0.02
	Nursing	56	3.7 ± 0.9	
When seeing other students have posted bullet screen comments, I would like to do the same.	Clinical medicine	55	4.1 ± 0.9	0.01
	Nursing	56	3.6 ± 1.2	
For me, posting bullet screen comments is privacy-preserving.	Clinical medicine	55	3.8 ± 1.0	0.86
	Nursing	56	3.8 ± 1.0	
I had enough ideas to contribute to the bullet board in class.	Clinical medicine	55	3.5 ± 0.9	0.003
	Nursing	56	3.0 ± 0.9	

### Perspectives on the education properties of bullet-screen interaction in medical and nursing students

We then evaluated the value of bullet screen for the improvement of learning outcomes in the face-to-face classroom. Almost three fourths (83/111 74.8%) (Table 4) of the students agreed that the interaction through the bullet screen could stimulate their in-depth thinking in class. However, among students majoring in nursing, the thought-provoking effect was poorer (Nursing 3.8 ± 0.9 Vs. Clinical medicine 4.2 ± 0.7, *P* = 0.01) (Table 4). In general, for approximately 32% of students, the bullet screen content was not useful for their learning (Table 4). Pre-clinical students tended to consider bullet-screen comments as an expression of support rather than as a method to make additional comments or to express a different opinion.

**Table 4:** Perspectives on the education properties of bullet-screen interaction in medical or nursing students

	Major	Number	Mean ± s.d.	*P* value
Bullet screen comments could stimulate my thinking of the course content.	Clinical medicine	55	4.2 ± 0.7	0.01
	Nursing	56	3.8 ± 0.9	
The bullet screen could help me to acquire a better understanding of the course content.	Clinical medicine	55	4.1 ± 0.8	0.12
	Nursing	56	3.8 ± 0.9	
When I wanted to give my approval for the content, I would post bullet screen comments.	Clinical medicine	55	3.4 ± 1.0	0.61
	Nursing	56	3.5 ± 1.0	
When I wanted to make additional remarks, I would post bullet screen comments.	Clinical medicine	55	2.9 ± 1.1	0.54
	Nursing	56	2.8 ± 1.0	
When I had a different idea in class, I could post bullet screen comments to express myself.	Clinical medicine	55	2.9 ± 1.0	0.48
	Nursing	56	3.0 ± 1.1	

### Perspectives on the interactive or entertainment properties of bullet-screen interaction in medical or nursing students

The combination of education and entertainment is popular in educational programming, which makes the process more engaging and enhances students’ motivation for listening and participating [16, 17]. Therefore, we also analysed the entertainment value and interactive effects of bullet screen. Ninety-eight (88.9%) students found that using bullet screen was interesting. However, only 76 (68.5%) students enjoyed the content of the bullet-screen comments. This finding may be due to the relatively serious classroom atmosphere. Additionally, students’ lack of ideas about course content may also lead to monotonous bullet-screen content. The effect on improving classroom interaction was better among students majoring in clinical medicine (4.3 ± 0.7) than those majoring in nursing (3.9 ± 0.7) (*P* = 0.003) (Table 5).

**Table 5:** Perspectives on the interactive or entertainment properties of bullet-screen interaction in medical or nursing students

	Major	Number	Mean ± s.d.	*P* value
The interactive process realized by the bullet screen was really interesting.	Clinical medicine	55	4.4 ± 0.8	0.96
	Nursing	56	4.4 ± 0.6	
Bullet screen could help improve the classroom interaction.	Clinical medicine	55	4.3 ± 0.7	0.003
	Nursing	56	3.9 ± 0.7	
The bullet screen comments were interesting.	Clinical medicine	55	4.0 ± 1.0	0.37
	Nursing	56	3.8 ± 0.9	

## Discussion

In this study, 111 pre-clinical students used the bullet screen in the classroom. Fifty-five of them majored in clinical medicine, while the remaining 56 majored in nursing. In medical education, we evaluated the essential features, instructional effectiveness, and entertainment value of bullet screens. We also investigated how specialty major influenced the variable characteristics. Our investigation results suggested a role for bullet screen in pharmacology education. In accordance with the Chinese social investigation, students found the interactive process realized by the bullet screen interesting (4.3 ± 0.7). They can immediately express their opinions on theoretical learning through this interaction (4.1 ± 0.8). Thus, the bullet screen can indeed improve classroom interaction (4.2 ± 0.7) and stimulate students’ in-depth thinking (3.9 ± 0.9). However, students still did not have enough ideas to participate, and their critical thinking ability needed further development. In addition, students majoring in nursing have even less subjective engagement. These phenomena informed teachers that a more elaborate course design is needed to further arouse students’ engagement and thinking. Overall, we determined that the bullet screen classroom was well liked by students and had a positive impact on improving classroom engagement and encouraging students’ self-expression. In view of all these benefits and high acceptance, we believe that the bullet screen can be popularized to a wider range of curricula. We have also identified possible improvements for better integration of this technology into classroom teaching.

During the COVID-19 pandemic, many online teaching platforms have emerged. Zoom is the preferred tool for videoconference [18]. Microsoft Teams group chat software has facilities to conduct quizzes and assignments, to evaluate assignments and to return them with comments, and to divide students into groups using teams and channels [19]. Online questionnaires are used to verify students’ learning outcomes. The bullet screen, despite its being solely used in China and Japan, did not conflict with the traditional Zoom meeting, and was applied during the Zoom videoconference. After scanning a QR code at the beginning of the class, students can post bullet screen comments at any time when listening to the teacher during the Zoom conference. Thus, the bullet screen could integrate smoothly with traditional online teaching platforms, but with more flexibility and interactivity: students could post comments whenever they wanted; the comments would fly across the screen so that they could be noticed at once; teachers did not need to create extra questionnaires; students could give feedback immediately on course content. Additionally, the coronavirus disease 2019 (COVID-19) pandemic has disrupted daily teaching activities. The abrupt transition to online courses, which lack interaction and communication, has presented additional challenges for educators [20]. In the case of students, a lack of sufficient planning, monitoring, and communication may lead to a reduction in motivation to learn at home [21]. Students are inclined to feel alone and disconnected from other students, and this mental obstacle has made remote education more difficult [22]. And bullet screen could help students to achieve self-expression and self-identification. That this auxiliary teaching tool may also help with alleviating the isolation and anxiety caused by online learning needs further investigation.

### Current problems and room for improvement in bullet screen classrooms

Although introducing the bullet screen into the classroom can improve classroom interaction, we found that the interaction was still insufficient. Students’ in-depth thinking can be stimulated by using a bullet screen, but it requires teachers’ guidance and question-and-answer design. Therefore, teachers need more prior knowledge and improvisation. At the other extreme, if teachers let students release their ideas with abandonment, the teaching rhythm will be seriously disturbed and the teaching objectives cannot be achieved. Hence, teachers should also properly grasp the limitations of bullet screen. In other words, the bullet screen classroom will require extra work for the teachers.

This classroom with bullet screen has its own scope of application. It is not suitable for courses containing numerous pieces of theoretical knowledge, which requires an elaborate explanation and leaves little time for divergent thinking. However, in a flipped classroom or a massive open online course (MOOC), the bullet screen may play a more important role. Students can explore, supplement, and criticize, etc. by themselves [14]. Additionally, we found that, in the courses containing more plot, unknown and exploration, for example, those concerning drug design and development, the bullet screen can inspire students’ creativity to the maximum and thus help them to explore the unknown. Whether bullet screen is more appropriate for these new classroom foramts in pharmacology education remains to be explored.

The bullet screen software needs some adjustments. Young people enjoy the anonymity of the traditional bullet screens, as their responses have few consequences, and people can feel free to express themselves without having to worry about judgement. However, the current bullet screen software used in the classroom did not hide the profile picture of students when showing comments. This has troubled many students, as they do not want to be recognized when making comments. In addition, the operation procedure of the software should be optimized for the ease of use of students. Some people may have concern about the safety of bullet screen, such as inappropriate comments being posted. We could set certain sensitive words to be blocked (e.g. abusive language) in the software background, thus the inappropriate comments could not be posted. Also, though the removal of profile pictures will make students masked in front of their classmates, teachers could examine the poster in the software background if inappropriate comments are submitted.

## Limitations

There are some limitations to consider when interpreting these results. First, this study is a single-centre analysis, which limits its broader generalizability to other institutions. Since Shanghai Jiao Tong University School of Medicine is one of the best medical colleges in China, whether the bullet screen can also work well in less prestigious colleges remains to be investigated. Second, the sample size was small and therefore we could not analyse students’ perception by, for example, age, gender, grade and online or face-to-face classroom. We only analysed the differences based on their specialty. This small sample size limits our ability to understand the potential effects on different student populations. Finally, we focused solely on students’ experience, but paid little attention to teachers’ opinions. Bullet screen is popular among young people, and whether older teachers can use and adapt to this technology needs further research. Teachers are the orienteer of the classroom. Further investigation of teachers’ evaluations is needed to better improve this model of teaching.

## Conclusions

We applied bullet screen technology to pharmacology education and evaluated its influence. Bullet screen was well received by students, and can conveniently increase classroom interaction. For instructional effectiveness, bullet screen might inspire students’ in-depth thinking. For entertainment value, the process of using bullet screen was interesting. The lack of ideas might lead to the relative differences found between medical and nursing students, which reminded us of the importance of better classroom guidance and the appropriate use of the bullet screen. Thus, the bullet screen may be popularized as an auxiliary teaching approach to promote interaction in the curriculum as well as in remote education. Its wider application and the difference between the students participating online and those in person need further investigations.

## Supplementary Information

**Additional file 1.** Questionnaire on the work of bullet screen in pre-clinical undergraduate pharmacology education.

## Data Availability

The datasets used and analysed during this study are available from the corresponding author on reasonable request.
