# Supplementary material for: Bullet screen in pre-clinical undergraduate pharmacology education: a survey study
Source: BMC Med Educ. 2022 Nov 28;22:813. doi: 10.1186/s12909-022-03906-6 (PMC9703395; doi:10.1186/s12909-022-03906-6)
Supplement: Supplementary file 1 — Additional file 1. Questionnaire on the work of bullet screen in pre-clinical undergraduate pharmacology education. [file 12909_2022_3906_MOESM1_ESM.docx]

**Questionnaire on the work of bullet screen in pre-clinical undergraduate pharmacology education**

Dear students,

In our pharmacology class, we used bullet screen method. The purpose of our research is to look into the performance of the bullet screen method in the classroom. There was no link between student names or identifying information and the replies made in this survey. This survey was done on a voluntary basis. We guarantee that your responses will be used only in this research. We hope you fill out the form honestly, based on your own opinions and thoughts.

Department of Pharmacology and Chemical Biology

Shanghai Jiao Tong University School of Medicine

**Appendix**

**Questionnaire on bullet screen classroom**

Q1 What is your gender?

- - Male
  - Female

Q2 What is your age?

Q3 What year are you currently enrolled in at the school of medicine?

- First year
- Second year
- Third year

|  | Strongly Disagree | Disagree | Neutral | Agree | Strongly Agree |
| --- | --- | --- | --- | --- | --- |
| It is not difficult for me to use the bullet screen function. | ○ | ○ | ○ | ○ | ○ |
| I could express my ideas in class through the bullet screen. | ○ | ○ | ○ | ○ | ○ |
| The bullet screen could help me to acquire a better understanding of the course content. | ○ | ○ | ○ | ○ | ○ |
| The interactive process realized by the bullet screen was really interesting. | ○ | ○ | ○ | ○ | ○ |
| The bullet screen comments were interesting. | ○ | ○ | ○ | ○ | ○ |
| When I had a different idea in class, I could post bullet screen comments to express myself. | ○ | ○ | ○ | ○ | ○ |
| When I wanted to make additional remarks, I would post bullet screen comments. | ○ | ○ | ○ | ○ | ○ |
| When I wanted to give my approval for the content, I would post bullet screen comments. | ○ | ○ | ○ | ○ | ○ |
| For me, posting bullet screen comments is privacy-preserving. | ○ | ○ | ○ | ○ | ○ |
| For me, posting bullet screen comments is convenient. | ○ | ○ | ○ | ○ | ○ |
| When seeing other students have posted bullet screen comments, I would like to do the same. | ○ | ○ | ○ | ○ | ○ |
| Bullet screen could help improve the classroom interaction. | ○ | ○ | ○ | ○ | ○ |
| Bullet screen comments could stimulate my thinking of the course content. | ○ | ○ | ○ | ○ | ○ |
| I was willing to participate in the bullet screen interaction. | ○ | ○ | ○ | ○ | ○ |
| The form of the bullet screen in class needs to be improved. | ○ | ○ | ○ | ○ | ○ |
| I had enough ideas to contribute to the bullet board in class. | ○ | ○ | ○ | ○ | ○ |
| It is beneficial to use bullet screen in class. | ○ | ○ | ○ | ○ | ○ |

* Five points were assigned for “strongly agree”, 4 points were assigned for “agree”, 3 points were assigned for “neutral”, 2 points were assigned for “disagree” and 1 point was assigned for “strongly disagree”.

Q4 Do you have any suggestions for the improvement of bullet screen in pharmacology teaching?
